# A new species of *Ocydromia* Meigen from China, with a key to species from the Palaearctic and Oriental Regions (Diptera, Empidoidea, Ocydromiinae)

**DOI:** 10.3897/zookeys.349.5473

**Published:** 2013-11-13

**Authors:** Yan Li, Mengqing Wang, Ding Yang

**Affiliations:** 1 Department of Entomology, China Agricultural University, Beijing 100193, China; 2 Key Laboratory of Integrated Pest Management in Crops, Ministry of Agriculture, Institute of Plant Protection, Chinese Academy of Agricultural Sciences, Beijing 100081, China

**Keywords:** Diptera, Empidoidea, *Ocydromia*, new species, China

## Abstract

Previously only one species of the genus *Ocydromia* Meigen was recorded from China. Here a second species of the genus from China, *Ocydromia shanxiensis* **sp. n.**, is reported. A key to the species of the genus from the Palaearctic and Oriental regions is presented.

## Introduction

The genus *Ocydromia* Meigen, 1820 is characterized by the following features: first flagellomere elliptical; arista long, supra-apical, bare and one-segmented; proboscis very short and fleshy; mesonotum almost bare, *acr* uni- or biserial; wing broad, 2 veins from broad discal cell; legs lacking distinct setae (Yang and Gaimari 2004). See Collin (1961) and Chvála (1983) for detailed descriptions of the genus. There are eight described species known from the world (Yang et al. 2007). Three species are found in the Palaearctic Region (Chvála 1983; Chvála and Kovalev 1989; Yang and Gaimari 2004), of which one is also distributed in the Nearctic Region (Melander 1965; Steyskal and Knutson 1981). Additionally, one species is found in the Neotropical (Rafael and Ale-Rocha 1990), two in the Afrotropical (Smith 1980), and two in the Oriental regions (Frey 1953; Smith 1975).

The biology of *Ocydromia* species is very interesting. *Ocydromia glabricula* (Fallén) displays obligate multilarviparity (Meier et al. 1999), with females flying over excrement and dropping larvae (Grunin 1953). Hobby and Smith (1962) described and illustrated the first instars. Chvála (1983) further suggested that *Ocydromia melanopleura* Loew is also viviparous based on finding dead first instars attached to the abdominal tips of dried specimens, and it seems likely to be characteristic for the genus.

Previously only one species, *Ocydromia xiaowutaiensis* Yang & Gaimari, was recorded from China (Yang and Gaimari 2004). In the present paper, a second species of the genus from China, *Ocydromia shanxiensis* sp. n., is reported. A third species, *Ocydromia unifasciata*, is known from Guizhou Province (Sinclair pers. comm.) (housed in CNC). A key to the species of *Ocydromia* from the Palaearctic and Oriental regions is presented.

## Material and method

The types of the new species are deposited in the Entomological Museum of China Agricultural University (CAU), Beijing. Basic terminology follows McAlpine (1981) and Steyskal and Knutson (1981). The following abbreviations are used for setae: acr – acrostichal, av – anteroventral, dc – dorsocentral, h – humeral, oc – ocellar, npl – notopleural, prsc – prescutellar acrostichal, psa – postalar, sa – supra-alar.

## Taxonomy

### Key to species of *Ocydromia* from Palaearctic and Oriental Regions

(modified from Yang and Gaimari 2004)

**Table:** 

1	Stigma long and narrow; mesonotum with no more than one spot	2
–	Stigma short and round; mesonotum with a median vitta and black spot above each wing base [male unknown] (Burma)	*Ocydromia stigmatica* Frey
2	Thorax mostly or entirely black in male, black or more or less yellow in female	3
–	Thorax bright brownish yellow with an oval black spot on anterior part of mesonotum [female unknown] (India)	*Ocydromia unifasciata* (Brunetti)
3	Thorax including pleuron black in both sexes; sense-organ of fore tibia with narrow hair brush pointed apically	4
–	Thorax mostly black or brownish yellow in male but more or less yellow in female; sense-organ of fore tibia with wide hair brush obtuse apically	5
4	Scutellum with one pair of distinct marginal setae (additional lateral marginal setae hardly differentiated from setulae along fringe); right surstylus strongly curved inwards, hypandrium truncated apically (Europe)	*Ocydromia melanopleura* Loew
–	Scutellum with three pairs of distinct marginal setae (apical pair longest); right surstylus weakly curved inwards, hypandrium not truncated apically (Palaearctic China)	*Ocydromia shanxiensis* sp. n.
5	Setulae on sense-organ of fore tibia soft and curved inwards apically; left and right epandrial lamellae fused basally by short narrow band, right surstylus without acute inner denticles apically, hypandrium wide basally but slightly narrowed towards tip [female unknown] (Palaearctic China)	*Ocydromia xiaowutaiensis* Yang et Gaimari
–	Setulae on sense-organ of fore tibia straight; left and right epandrial lamellae fused basally by long narrow band, right surstylus with two acute inner denticles apically, hypandrium narrow basally and wide apically	*Ocydromia glabricula* (Fallén)

### Ocydromia shanxiensis sp. n.

http://zoobank.org/1D91ED0D-EF45-493A-B8E3-220A3133D5DE

http://species-id.net/wiki/Ocydromia_shanxiensis

Figs 1 –9

#### Diagnosis.

Thorax polished black in both sexes; female abdomen partly yellow. Scutellum with three pairs of marginal setae. Legs mostly blackish, except coxae and trochanters yellow, and femora brownish yellow except apical portions of fore and mid femora brown and apical portion of hind femur brownish. Sense-organ of fore tibia with narrow hair brush pointed apically. Hypandrium distinctly longer than wide, with obtuse apex.

#### Description.

**Figure 1.**
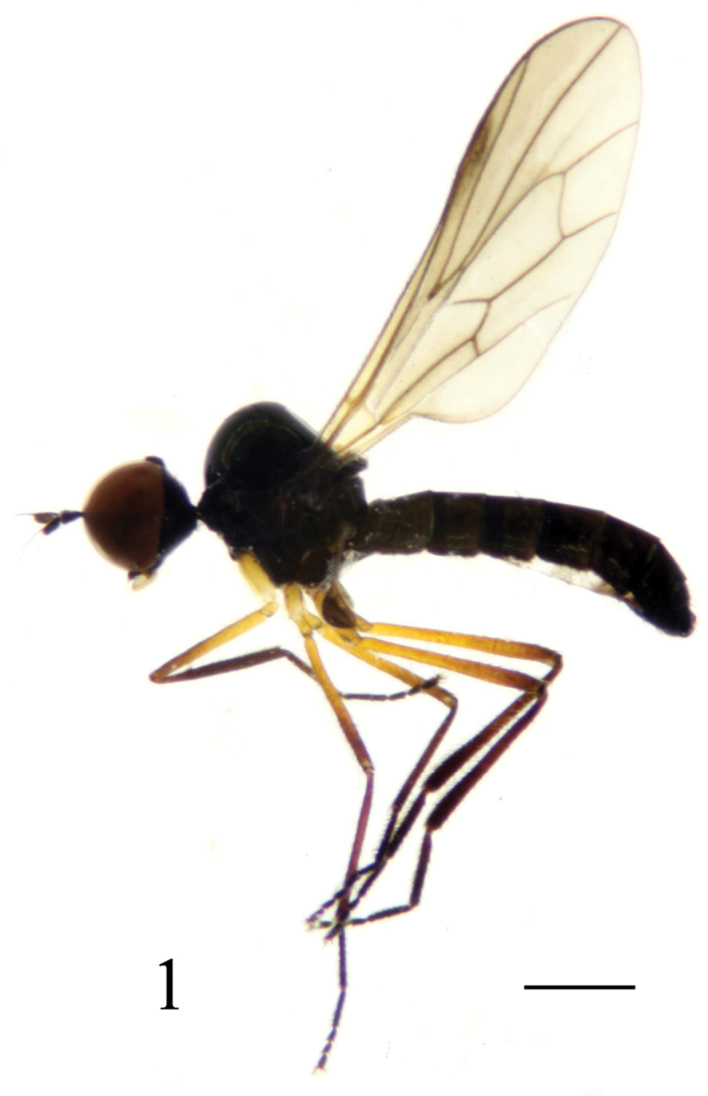
*Ocydromia shanxiensis* sp. n., male adult. Scale bar 1 mm.

Male (Fig. 1). Body length 3.1–3.2 mm, wing length 2.8–2.9 mm.

Head black with gray pollinosity. Eyes contiguous on frons, brownish, with upper facets slightly enlarged; face linear. Setulae and setae on head black, posteroventral setulae dark yellow. Ocellar tubercle distinct with 2 long oc and 2 very short posterior setulae. Antenna black; pedicel with circlet of black subapical setulae; first flagellomere elliptical, 2.0 times longer than wide, minutely pubescent; arista long (2.7–2.8 times as long as first flagellomere), supra-apical, bare, one-segmented and black. Proboscis short, mostly brownish yellow, with black setulae; palpus black with black setulae and 2 thin black setae.

Thorax mostly polished black except postalar callus dark brownish yellow; mesonotum with narrow mid-lateral area and scutellum with gray pollinosity. Setulae on thorax blackish, setae weak and black; setulae on mesonotum sparse; humerus with 3–4 setulae, without h; 2 npl; acr and dc uniseriate and hair-like; 1 sa; 1 psa; 1 prsc; scutellum with short dense pubescence and 3 pairs of sc (apical pair distinctly longer than lateral pairs). Legs mostly blackish, except coxae and trochanters yellow, and femora brownish yellow except apical portions of fore and mid femora brown and apical portion of hind femur brownish. Setulae and setae on legs blackish, setae weak; coxae with yellow setulae and setae, hind femur with hair-like av slightly longer than femur thickness. Sense-organ of fore tibia with narrow hair brush pointed apically (Fig. 4). Hind tibia distinctly thickened apically; hind tarsomere 1 slightly thickened, slightly shorter than tarsomeres 2–5. Wing (Fig. 3) hyaline, tinged gray; stigma dark brown, about 1/4 as long as cell r_1_; veins dark brown. Squama dark brown with dark brown setulae. Halter dark brown.

Abdomen slightly curved downward and polished blackish; venter with gray pollinosity. Setulae and setae on abdomen blackish; tergites 1–2 with dark yellow lateral setulae, sternites 1–2 with dark yellow setulae.

Male genitalia (Figs 5–9). Left and right epandrial lamellae fused basally by narrow band. Left epandrial lamella narrow in dorsal view; left surstylus finger-like, strongly curved inwards. Right epandrial lamella wide basally in dorsal view; right surstylus weakly curved inwards with acute apex; left and right cerci subequal in length and obtuse apically. Hypandrium distinctly longer than wide, with obtuse apex. Two branches of bifid appendage at tip of phallus equally long but unequally stout.

**Figure 2.**
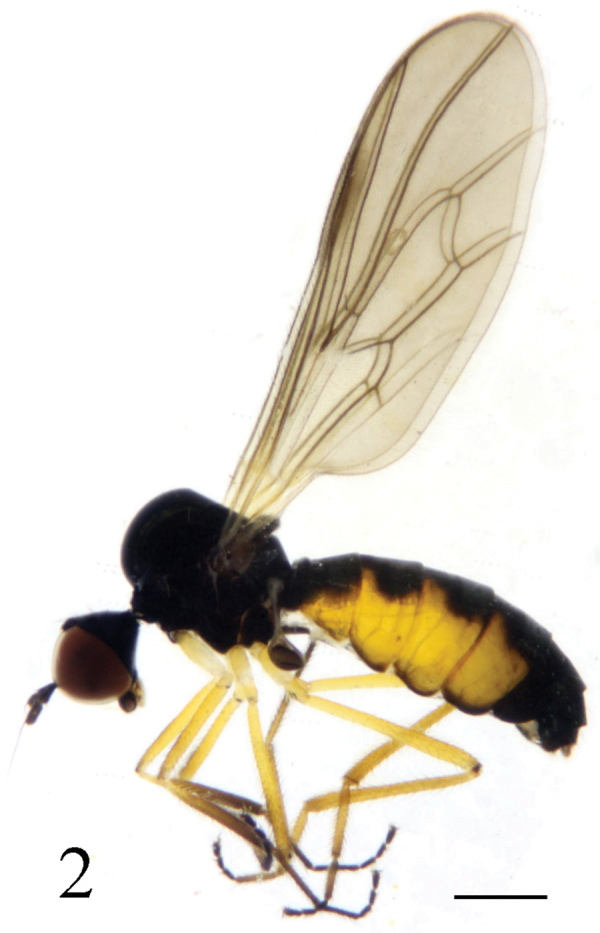
*Ocydromia shanxiensis* sp. n., female adult. Scale bar 1 mm.

Female (Fig. 2). Body length 3.1–3.4 mm, wing length 3.6–3.7 mm. Similar to male, but abdomen distinctly swollen, tergites 2–5 yellow laterally and tergite 6 sometime yellow at antero-lateral portion. Legs dark yellow except coxae and trochanters yellow, fore tibia and tarsus blackish; mid and hind tibiae dark brownish yellow, tarsi dark brown except tarsomere 1 dark yellow and tarsomere 2 brownish.

**Figures 3–4.**
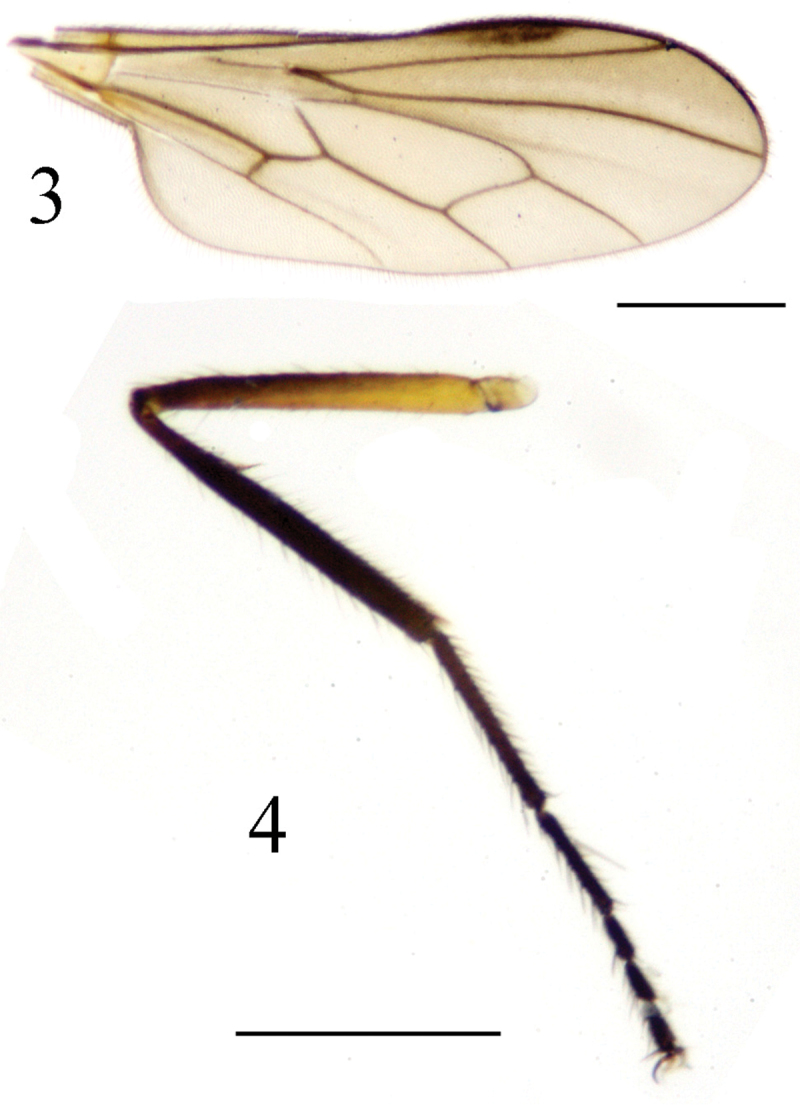
*Ocydromia shanxiensis* sp. n. (male) **3** wing **4** fore tibia, lateral view. Scale bar 1 mm.

**Figures 5–9.**
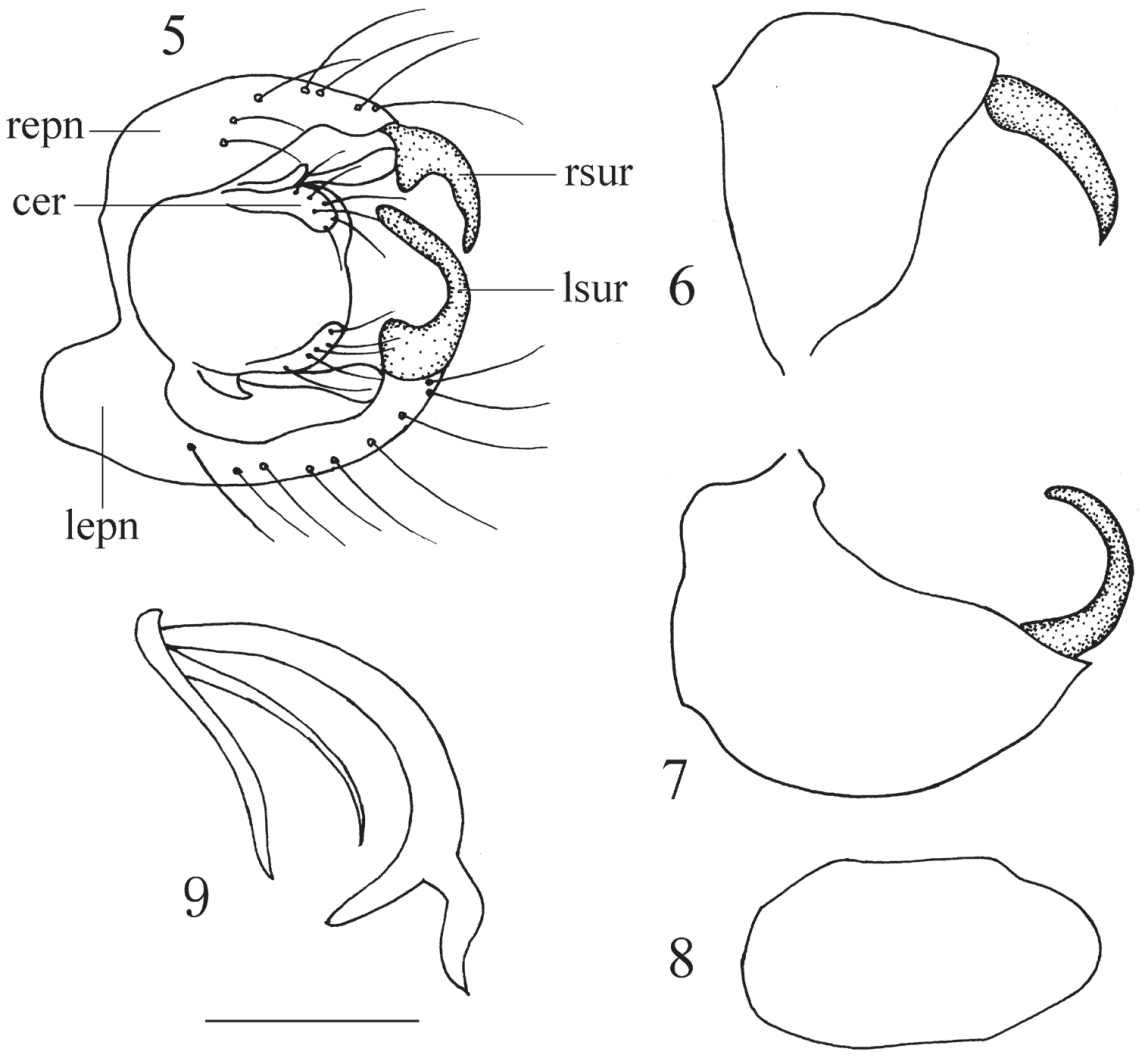
*Ocydromia shanxiensis* sp. n. **5** male genitalia, dorsal view **6** right epandrial lamella and surstylus, lateral view **7** left epandrial lamella and surstylus, lateral view **8** hypandrium, ventral view **9** phallus, lateral view. Abbreviations: cer = cercus; lepn = left epandrial lamella; lsur = left surstylus; repn = right epandrial lamella; rsur = right surstylus. Scale bar = 0.1 mm.

#### Type material.

Holotype: male, China: Shanxi Province, Yicheng, Yishan, Dahe, 2012.VII.24, Zhenghua Zhang (in 75% alcohol, deposited in CAU). Paratypes: 3 males, 4 females, same data as holotype (in 75% alcohol, deposited in CAU); 1 male, 1 female, China: Shanxi Province, Yicheng, Yishan, Dahe, 2012.VII.23, Chen Wang (in 75% alcohol, deposited in CAU).

#### Distribution.

China (Shanxi).

#### Remarks.

The new species is similar to the European species *Ocydromia melanopleura*, but may be distinguished from the latter by the scutellum with three pairs of distinct marginal setae (apical pair longest), right surstylus weakly curved inwards, and hypandrium obtuse apically. In *Ocydromia melanopleura*, the scutellum has only one pair of distinct marginal setae, the right surstylus is strongly curved inwards, and the hypandrium is truncated apically (Chvála, 1983).

#### Etymology.

The species is named after the type locality Shanxi.

## Supplementary Material

XML Treatment for Ocydromia shanxiensis

## References

[B1] Chvála M (1983) The Empidoidea (Diptera) of Fennoscandia and Denmark. II. General part. The families Hybotidae, Atelestidae and Microphoridae. Fauna Entomologica Scandinavica 12: 1-279

[B2] Chvála M Kovalev VG (1989) Family Hybotidae. In: Soós Á Papp L (Eds) Catalogue of Palaearctic Diptera. Volume 6 Elsevier Science Publishers & Akadémiai Kiadó, Amsterdam & Budapest, 174-227

[B3] Collin JE (1961) Empididae. In: Verrall GH (Ed) British Flies, Volume 6 Cambridge University Press, London, 782 pp.

[B4] Frey R (1953) Studien über ostasiatische Dipteren. II. Hybotinae, Ocydromiinae, *Hormopeza* Zett. Notulae Entomologicae 33: 57-71

[B5] Grunin KY (1953) Viviparity in coprobionts in the order Diptera. Trudy Zoologischeskogo Instituta, Akademiya Nauk, SSSR 13: 387-389 [In Russian]

[B6] Hobby BM Smith KGV (1962) The larva of the viviparous fly *Ocydromia glabricula* (Fln.) (Dipt., Empididae). Entomologist’s Monthly Magazine 98: 49-50

[B7] McAlpine JF (1981) Morphology and Terminology - Adults. In: McAlpine JF et al. (Coords) Manual of Nearctic Diptera, Volume 1 Research Branch, Agriculture Canada, Ottawa. Monograph 27: 9–63

[B8] Meigen JW (1820) Systematische Beschreibung der bekannten europäischen zweiflügeligen Insekten. Zweiter Theil. F.W. Forstmann, Aachen, 363 pp.

[B9] Meier R Kotrba M Ferrar P (1999) Ovoviviparity and viviparity in the Diptera. Biological Reviews 74: 199-258.10.1017/S0006323199005320

[B10] Melander AL (1965) Family Empididae. In: Stone A et al. (Eds) A Catalog of the Diptera of America North of Mexico. Agriculture Handbook No. 276, Washington, D.C., 446–481

[B11] Rafael JA Ale-Rocha R (1990) Primeiro registro do gênero *Ocydromia* Meigen na Região Neotropical e descrição de *O. amazonica*, sp. n. (Diptera, Empididae, Ocydromiinae). Revista Brasileira de Entomologia 34: 739-741

[B12] Smith KGV (1975) Family Empididae. In: Delfinado MD Hardy DE (Eds) A Catalog of the Diptera of the Oriental Region, Volume 2 The University Press of Hawaii, Honolulu, 185-211

[B13] Smith KGV (1980) 32. Family Empididae. In: Crosskey R (Ed) Catalogue of the Diptera of the Afrotropical Region. British Museum (Natural History), London, 431-442

[B14] Steyskal GC Knutson LV (1981) Empididae In: McAlpine JF et al. (Coords) Manual of Nearctic Diptera, Volume 1 Research Branch, Agriculture Canada, Ottawa Monograph 27: 607-624

[B15] Yang D Gaimari SD (2004) Notes on the species of the genus *Ocydromia* Meigen from China (Diptera: Empididae). Pan-Pacific Entomologist 80(1–4): 62-66

[B16] Yang D Zhang KY Yao G Zhang JH (2007) World catalog of Empididae (Insecta: Diptera). China Agricultural University Press, Beijing, 599 pp.

